# Contrasting compositions of sitting, standing, stepping, and sleeping time: associations with glycaemic outcome by diabetes risk

**DOI:** 10.1186/s12966-021-01209-5

**Published:** 2021-12-04

**Authors:** Christian J. Brakenridge, Genevieve N. Healy, Parneet Sethi, Alison Carver, John Bellettiere, Agus Salim, Sebastien F. M. Chastin, Neville Owen, David W. Dunstan

**Affiliations:** 1 Baker Heart and Diabetes Institute, 99 Commercial Rd, Melbourne, VIC 3004 Australia; 2 Mary Mackillop Institute for Health Research, Australian Catholic University, Melbourne, VIC Australia; 3 School of Public Health, The University of Queensland, Brisbane, QLD Australia; 4 Herbert Wertheim School of Public Health and Longevity Science, University of California San Diego, La Jolla, CA USA; 5 School of Population and Global Health, The University of Melbourne, Melbourne, VIC Australia; 6 School of Health and Life Sciences, Glasgow Caledonian University, Glasgow, UK; 7 Department of Movement and Sports Science, Ghent University, Ghent, Belgium; 8 Centre for Urban Transitions, Swinburne University of Technology, Melbourne, VIC Australia

**Keywords:** Time-use, Diabetes risk, Glycaemic control, Sedentary behaviour

## Abstract

**Background:**

Recent evidence suggests that prolonged sitting and its adverse impact on glycaemic indicators appear to be proportional to the degree of insulin resistance. To investigate this finding in a free-living context, we aimed to examine associations of device-measured 24-h time-use compositions of sitting, standing, stepping, and sleeping with fasting glucose (FPG) and 2 h post-load glucose (2hPLG) levels, and to examine separately the associations with time-use compositions among those at lower and at higher risk of developing type 2 diabetes.

**Methods:**

Cross-sectional analyses examined thigh-worn inclinometer data (activPAL, 7 day, 24 h/day protocol) from 648 participants (aged 36-80 years) at either lower (< 39 mmol/mol; < 5.7% HbA1c) or higher (≥39 mmol/mol; ≥5.7% HbA1c) diabetes risk from the 2011-2012 Australian Diabetes, Obesity and Lifestyle study. Multiple linear regression models were used to examine associations of differing compositions with FPG and 2hPLG, with time spent in each behaviour allowed to vary up to 60 min.

**Results:**

In general, the associations with the FPG within the time-use compositions were small, with statistically significant associations observed for sitting and sleeping (in the lower diabetes risk group) and standing (in higher diabetes risk group) only. For 2hPLG, statistically significant associations were observed for stepping only, with findings similar between lower (β = − 0.12 95%CI:−0.22, − 0.02) and higher (β = − 0.13 95%CI:−0.26, − 0.01) risk groups. Varying the composition had minimal impact on FPG; however 1 h less sitting time and equivalent increase in standing time was associated with attenuated FPG levels in higher risk only (Δ FPG% = − 1.5 95%CI: − 2.4, − 0.5). Large differences in 2hPLG were observed for both groups when varying the composition. One hour less sitting with equivalent increase in stepping was associated with attenuated 2hPLG, with estimations similar in lower (Δ 2hPLG% = − 3.8 95%CI: − 7.3, − 0.2) and higher (Δ 2hPLG% = − 5.0 95%CI: − 9.7, − 0.0) risk for diabetes.

**Conclusions:**

In middle-aged and older adults, glycaemic control could be improved by reducing daily sitting time and replacing it with stepping. Standing could also be beneficial for those at higher risk of developing type 2 diabetes.

## Background

Clinical practice guidelines for the prevention of type 2 diabetes emphasise lifestyle management as the first priority for those identified with elevated risk [1]. A primary focus has been on promoting regular participation in moderate-to-vigorous intensity physical activity. More recently, addressing sedentary (sitting) time has been included in guidelines [2] based on emerging observational and acute experimental evidence regarding the detrimental relationships of high volumes of time spent sitting with risk for type 2 diabetes [3] and its precursors [4], and the potential benefits of replacing sitting time with physical activity and/or standing [5–7].

Consideration of 24 h time-use as a composition of distinct yet competing activities (sitting, standing, stepping, and sleep) is increasingly being adopted in observational research to examine associations with health outcomes and risk biomarkers [8–10]. This has been made possible by the use of continuously-worn activity-monitor devices that collect time- and date-stamped information, enabling the 24 h period to be categorised entirely into the sum of time spent in different behaviours. This has led to the creation of integrated movement guidelines, that have made recommendations on how to best utilise the 24 h for greatest health benefit [11]. There have been numerous studies that have suggested that time spent sitting [7], standing [12], in physical activities [13], and in sleep [14] can have distinct associations with glucose outcomes. However, the majority of these studies did not differentiate by posture (instead, they did so using device acceleration thresholds). Nor have they considered these behaviours as interrelated exposures [15], where spending time in one behaviour will necessarily mean less time undertaken in the remaining behaviours within the same 24 h period.

Findings from recent laboratory-based trials suggest that the detrimental impacts of prolonged sitting time on blood glucose may be proportional to the degree of underlying insulin resistance [16], but it is not known if this is manifested in the free-living context. Furthermore, it is unclear if changing sitting, standing, and activity levels have differential impact depending on underlying risk for developing type 2 diabetes. This has important implications for the tailoring of public health advice, and for clinical practice guidelines targeting vulnerable populations.

To address these evidence gaps, compositional data analyses (CoDA) were used to examine the associations with fasting plasma glucose (FPG) and 2 h post-load glucose (2hPLG) of device-measured components of 24 h time-use (sitting, standing, stepping, and sleeping) in the free-living context in middle-aged and older Australian adults. Compositions with varying time spent between components were compared between those at lower and higher risk for type 2 diabetes.

## Methods

### Participants and setting

The Australian Diabetes, Obesity and Lifestyle Study (AusDiab) baseline study methods and response rates are described in detail elsewhere [17]. In brief, the baseline study was a national population-based survey of 11,247 adults aged ≥25 years in 1999-2000. A stratified cluster sampling approach was undertaken, with strata selected based upon the six states and the Northern Territory of Australia. Subsequent follow-ups occurred in 2004–2005 (*n* = 6400) and 2011-2012 (*n* = 4614). At the 2011/2012 follow-up, a sub-sample of participants were invited to wear an activity monitor as described elsewhere [7]. Eligible participants (ambulatory, and not pregnant) were recruited daily in a consecutive manner until either a quota was reached (*n* = 5) or no more monitors were available. Of the 1014 approached, 782 consented to wearing the monitor, and 741 wore the monitor for at least one valid day. For these analyses, the following exclusions were applied: those with less than four valid monitor-wear days (*n* = 21), those with known diabetes (diagnosed by physician and taking hypoglycaemic medication or insulin) at the assessment (*n* = 37); those pregnant (*n* = 2); and those with missing covariates (*n* = 33). A small number of participants missed assessment of 2hPLG (*n* = 8) and FPG (*n* = 1) in the oral glucose tolerance test (OGTT) assessment. Thus, the final cross-sectional study was conducted with 647 for the FPG analysis and 640 for the 2hPLG analysis. The sample was stratified into two groups according to their HbA1c (glycated hemoglobin) levels: lower risk for diabetes (< 39 mmol/mol, < 5.7% HbA1c), and higher risk (≥39 mmol/mol, ≥5.7 HbA1c). This was conducted a priori and based on experimental research suggesting that behaviours are associated with varied glucose outcomes depending on the degree of dysmetabolism [16]. Stratification is also in accordance with the American Diabetes Association’s diagnosis classification for prediabetes [18].

### Data collection

On the day of recruitment, participants followed standard protocols as per the main AusDiab study procedures [19]. Following an overnight fast (minimum 8 h), participants underwent biochemical and anthropometric assessments and completed a series of questionnaires at their local testing site. FPG and HbA1c were taken initially, followed by a standard 2 h 75-g OGTT [20]. The activity monitor was put on either on the day of the assessment or the following day. Questionnaires (self-completed and interviewer administered) were used to collect data on confounding variables.

### Device-measured sitting, standing, stepping, and sleep time

The time-use composition was derived from measurements using the activPAL3 activity monitor (PAL technologies Limited, Glasgow, UK; version 6.4.1); this device has been shown to be accurate and reliable for use with adults and older adults [21]. Each monitor was initialised with the default settings (20 Hz) and waterproofed by covering it in a nitrile sleeve and then encased in transparent Hypafix. It was then secured anteriorly to the participant’s right thigh at the approximate midline. Participants were instructed to wear the monitor continuously for seven consecutive days (24 h/day, keeping the device attached for showering/bathing), and to record in a standardised diary their sleep and monitor removal time (if it did occur). The device was mailed back to the AusDiab research staff in a reply-paid envelope at completion. Monitor data were processed using SAS 9.3 (SAS institute Inc., Cary, NC, USA). An invalid wear day was considered to be when monitor wear time was less than 80% of waking hours, or less than 10 h if the participant’s diary was missing sleep and wake times. Invalid wear days were excluded from data analysis. If sleep and wake times were not reported in the diary they were estimated using an automated algorithmic method [22], which demonstrated almost perfect agreement for most participants (median kappa 0.94 in 88% of participants). Sleep time was deduced by subtracting all waking behaviour and unworn monitor time from 24 h. Across all valid days, the average time spent in sitting, standing, stepping, and sleeping within a 24 h period was calculated.

### Glycaemic measures

Blood samples were collected via venipuncture with whole blood collected into fluoride-oxalate containing tubes for the analysis of plasma glucose, and EDTA containing tubes for the analysis of HbA1c. All blood specimens were centrifuged on-site in order to separate plasma, which was then immediately aliquoted for testing and storage. Storage entailed either the immediate transport of the sample to a central independent laboratory (Healthscope Pathology) or to the site freezer where it was kept at − 20 °C and subsequently at − 80 °C within 1 to 2 weeks after collection. HbA1c was measured with liquid chromatography method (Bio-Rad Variance Hemoglobin Testing System; Bio-Rad, Hercules Ca, USA). FPG and 2hPLG were measured by the hexokinase method using a Siemens Advia 2400 (Siemens AG, Munich, Germany).

### Other measures

Backwards elimination was performed on a set of confounders previously identified in the activity monitor subsample (covariate elimination where *p* > 0.2) [7]. Variables excluded were contraceptive medication, ethnicity, employment status (blue collar, white collar, unemployed), occupation type (manager, professional, technician, service worker, clerical worker, sales worker, machinery, labourer worker, labourer, never worked), fibre intake, and marital status. Smoking status and menopausal status were excluded, however given their potential for modifying glucose metabolism; they were added back into the confounder adjusted models. Confounders used for all models included: age, menopausal status (self-reported pre, peri, post-menopausal status, or male), education attainment, income category, smoking category, depression status [23], diet quality [24], energy intake, alcohol and calcium intake. These analyses did not account for confounding by adiposity as it was considered on the causal pathway and thus a mediator of the relationships. A separate analysis with adjustment by waist circumference revealed similar magnitude and direction for all relationships.

### Statistical analyses

Analyses were conducted using Stata 14.2 (StataCorp LP, College Station, TX, USA) and R version 3.6.1. Group characteristics were compared using analysis of variance for continuous variables, and by chi-square tests for categorical variables. In the linear regressions, glucose outcomes were log transformed which improved normality of residuals. Multicollinearity among confounders were assessed using variance inflation factor (VIF) methods, all models had VIF values below 2.5.

The CoDA method procedure has been described in detail previously [9, 25]. The 24 h day (1440 min) examined was finitely comprised of sitting, standing, stepping, and sleeping time-use components. Daily totals of all activities were calculated into geometric means per diabetes risk group using Aitchison’s perturbation method (“acomp” function in R package: Compositions). Means were transformed into isometric log ratios and compared between higher and lower risk groups. In order to explain significant group difference, geometric means were first computed as a log ratio (higher/lower risk), and then bootstrapped (as described by Gupta et al. [26]) to calculate the percentage difference (difference between two log ratios) between the two geometric means with upper and lower limits of 95% confidence intervals. Overall group difference was determined with Hotelling’s test (R package: “Hotelling”).

The compositional modeling employs isometric log ratios (ilr) of the behaviour components. In brief, the outcome (log glucose) is dependent on the sum of composition isometric log ratios and covariates through a regression model.

$$E\ \left(y\ | ilr\right)={\beta}_0+{\beta}_1 ilr 1+{\beta}_2 ilr 2+{\beta}_3 ilr 3+ effect\ of\ other\ covariates$$

Where ilr1, ilr2 and ilr3 are the coordinates of the ilr-transformed composition. The coefficient β_1_ is the main interest here as it reflects the effect of time spent in one behavior relative to the other three. For example, to assess the effect of time spent sitting relative to stepping, standing, and sleeping, we would use ‘sitting’ as the reference behavior when performing the ilr transformation and compute ilr1 as:

$$\mathrm{ilr}1:\kern0.5em \left( sitting\ vs. standing, stepping, and\ sleeping\right)=\sqrt{\left( 3/ 4\right)ln\left( sitting\right)}/\sqrt[ 3]{\left( stand\ast step\ast sleep\right)}$$

To assess the interaction with diabetes risk, an interaction between ilr-transformed variables (ilr1, ilr2, ilr3) and diabetes risk group was added to the model and the interaction coefficient with ilr1 was examined for statistical significance. To test an outcome’s association with relative time spent in the other behaviours, the ilr-transformed variable was recalculated using that behaviour as reference and the above procedures were repeated. For all behaviours, associations were tested in unadjusted and confounder-adjusted models. The models were then used to estimate the expected log FPG and 2hPLG values with set compositions.

New compositions were made by adding and subtracting 15, 30, 45, and 60 min from the geometric mean values. The difference (delta) between the estimated log glucose value of the new composition and the estimated value of the geometric mean composition was calculated using R package “deltacomp” [10, 27]. Confidence intervals were determined using the standard error of the delta estimate. The estimated differences were then back-transformed and presented as percentage difference from the original glucose value. For each of the behaviours, there was no requirement to adjust them from zero time as every participant participated in at least 1 min in each of the behaviours. All hypothesis testing was two-tailed and the type I error for all statistical analyses were set at 5%.

## Results

### Characteristics of participants

Table 1 describes the characteristics of participants with lower and higher diabetes risk. Compared to the lower risk, the higher risk participants were more likely to be older, and to include post-menopausal women, and earn less. There were no significant differences in behaviours between the lower risk and higher risk for smoking, energy intake, or dietary quality. Alcohol intake was greater in the lower risk (15.5 g) compared to the higher risk (11.5 g).

**Table 1:** Sample characteristics stratified by lower and higher risk for diabetes

	Diabetes Risk	
	Lower Risk (*n* = 376)	Higher Risk (*n* = 272)
HbA1c, *mmol/mol 95% CI*	36 (36–37)	41 (41–41)*
HbA1c%, *95 CI*	5.4 (5.4–5.5)	5.9 (5.9–5.9)*
FPG, *mmol/L (sd)*	5.2 (0.5)	5.5 (0.9)*
2hPLG, *mmol/L (sd)*	5.2 (1.3)	6.0 (2.3)*
Socio-demographic		
Age, *years (sd)*	56.0 (9.8)	60.2 (9.3)*
Women, *n (%)*	198 (52.7%)	165 (60.7%)
Education, *n (%)*		
High school or less	93 (24.7%)	95 (34.9%)*
Technical / Vocational	184 (48.9%)	116 (42.6%)*
Bachelor degree or higher	99 (26.3%)	61 (22.4%)*
Income, *n (%)*		
No income, or not reported	22 (5.9%)	17 (6.2%)
$1-39,999 per year	65 (17.3%)	66 (24.3%)
$40,000-79,999 per year	91 (24.2%)	71 (26.1%)
≥ $80,000 per year	198 (52.7%)	118 (43.4%)
Menopause, *n (%) women*		
Post-menopausal	88 (44.4%)	117 (70.9%)*
Peri-menopausal	37 (18.7%)	20 (12.1%)*
Pre-menopausal	73 (36.9%)	28 (17.0%)*
Known depressive symptoms, *n (%)*^a^		
Yes	23 (6.1%)	29 (10.7%)
Behaviour		
Smoking status, *n (%)*^b^		
Current smoker	28 (7.4%)	18 (6.6%)
Ex-smoker	139 (37.0%)	97 (35.7%)
Non-smoker	209 (55.6%)	157 (57.7%)
Dietary Intake		
Energy, *mcal/day*	1.7 (0.65)	1.7 (0.67)
Dietary quality score	65.7 (12.4)	67.2 (12.6)
Alcohol*, g/day*	15.5 (19.1)	11.5 (15.3)*
Calcium, *g/day*	0.9 (0.3)	0.9 (0.3)

Table displays mean (standard deviation), or sample n (%)

*Indicates significant difference between stratified groups with *p <* 0.05

^a^Known depressive symptoms indicated when CESD score ≥ 10

^b^Smoking status: Current smoker: smokes now, and ≥ 100 cigarettes in lifetime, Ex-smoker: does not currently smoke and ≥ 100 cigarettes in lifetime, Non-smoker: smoked < 100 cigarettes in lifetime and does not currently smoke

### Geometric means for the 24 h day

Table 2 shows the geometric means of sitting, standing, stepping, and sleeping for the lower and higher risk participants. For both the lower and higher risk, sitting occupied the largest proportion of the day and stepping the smallest. When considering group geometric means and variance intervals comparing the two risk groups, all percentage differences intersected zero; therefore, there were no statistically-significant differences between groups. The comparisons using Hotelling’s test confirmed no statistically significant difference between the overall compositions with *p*-value > 0.05.

**Table 2:** Geometric means of behaviours in those with lower and higher risk for diabetes

Diabetes Risk	Lower Risk (*n* = 376)	Higher Risk (*n* = 272)	Percentage Difference (95% CI)^b^
Geometric means, *mins/1440*^a^			
Sitting	526.4 (36.6%)	530.7 (36.9%)	0.49% (−4.44, 5.41)
Standing	287.1 (19.9%)	288.5 (20.0%)	− 3.90% (−9.36, 1.59)
Stepping	120.6 (8.4%)	116.0 (8.1%)	−0.21% (− 2.20, 1.84)
Sleeping	505.8 (35.1%)	504.8 (35.1%)	0.80% (−2.56, 4.08)

^a^Geometric means expressed as minutes conducted within a 1440 min composition (percentage rounded to complete number)

^b^Percentage difference refers to the log ratio difference between each behaviour per group converted into percentage. Positive estimated difference indicates that the higher risk has a greater level of the given component; negative estimated difference indicates the lower risk has a greater level of the given component. Percentage difference and 95% confidence intervals were determined with bootstrapping. Behaviours with confidence intervals that intersect zero should be considered to not differ by diabetes risk group

### Compositional linear regression modelling

The associations of log glucose outcomes with time spent in each behaviour (and consequently less time in remaining behaviours) are presented for both the lower and higher diabetes risk participants unadjusted and confounder adjusted in Table 3. In the confounder adjusted model, for lower risk all associations with FPG were weak, with statistically significant associations observed for sitting (β = 0.04 95%CI: 0.00, 0.08) and sleeping (β = − 0.06 95%CI: − 0.12, − 0.00) only. Associations with FPG were also weak in the higher risk, with the only statistically significant association being with standing time (β = − 0.07 95%CI: − 0.12, − 0.01). However, the direction of the relationships of standing and sleeping with FPG were opposite between groups (*p* < 0.01 for interaction); this was the only statistically significant interaction by diabetes group observed. For 2hPLG, statistically significant associations were observed for stepping only, with associations similar for the lower (β = − 0.12 95%CI: − 0.22, − 0.02) and higher (β = − 0.13 95%CI: − 0.26, − 0.01) risk groups. Notably, there was a positive association with 2hPLG and sleeping time in the high risk group (β = 0.17 95%CI: − 0.04, 0.39); however, this finding did not reach statistical significance.

**Table 3:** Associations of behaviours with glucose biomarkers overall and in lower and higher risk for diabetes

Behaviours (γ1)	FPG				2hPLG			
	Overall β (95% CI)	Lower Risk β (95% CI)	Higher Risk β (95% CI)	*p*-value int.^a^	Overall β (95% CI)	Lower Risk β (95% CI)	Higher Risk β (95% CI)	*p*-value int.^a^
Unadjusted model								
Sitting	0.05* (0.01, 0.08)	0.06* (0.02, 0.10)	0.03 (- 0.04, 0.10)	0.444	0.02 (- 0.07, 0.12)	0.04 (- 0.07, 0.15)	0.00 (- 0.16, 0.16)	0.647
Standing	-0.05* (- 0.08, -0.02)	-0.01 (- 0.05, 0.02)	-0.10* (- 0.15, -0.04)	0.012*	-0.05 (- 0.14, 0.04)	-0.07 (- 0.18, 0.04)	-0.05 (- 0.19, 0.09)	0.815
Stepping	-0.02 (- 0.04, 0.01)	0.02 (- 0.02, 0.05)	-0.03 (- 0.08, 0.02)	0.133	-0.14* (- 0.22, - 0.07)	-0.07 (- 0.16, 0.03)	-0.18* (- 0.30, - 0.06)	0.138
Sleeping	0.02 (- 0.03, 0.07)	-0.06* (- 0.12, - 0.00)	0.09* (0.01, 0.18)	0.002*	0.17* (0.04, 0.30)	0.10 (- 0.06, 0.26)	0.24* (0.03, 0.45)	0.298
Confounder adjusted model								
Sitting	0.04* (0.01, 0.08)	0.04* (0.00, 0.08)	0.03 (-0.03, 0.10)	0.479	0.02 (-0.08, 0.11)	0.00 (-0.12, 0.11)	0.01 (- 0.15, 0.18)	0.791
Standing	-0.03 (- 0.06, 0.01)	0.02 (- 0.01, 0.06)	-0.07* (- 0.12, - 0.01)	0.002*	-0.03 (- 0.11, 0.06)	0.01 (- 0.10, 0.13)	-0.05 (- 0.20, 0.09)	0.972
Stepping	-0.02 (- 0.05, 0.01)	-0.01 (- 0.04, 0.03)	-0.03 (- 0.08, 0.02)	0.396	-0.12* (- 0.20, - 0.05)	-0.12* (- 0.22, - 0.02)	-0.13* (- 0.26, - 0.01)	0.255
Sleeping	0.00 (- 0.05, 0.05)	-0.06* (- 0.12, - 0.00)	0.07 (- 0.02, 0.15)	0.002*	0.13* (0.00, 0.26)	0.11 (- 0.06, 0.27)	0.17 (- 0.04, 0.39)	0.378

Beta coefficients presented with 95% confidence interval (CI). Coefficient corresponds to association of time spent in the behaviour with log glucose outcome

All confounder adjusted models adjusted for age, menopausal status (pre, peri, post-menopausal, male), education attainment, income category, smoking category, depression, diet quality, energy intake, alcohol, and calcium intake

^a^*p*-value interaction indicates where association is statistically different by diabetes risk

*Indicates a statistically significant association, and statistically significant interaction by diabetes risk using *p <* 0.05 in two tailed analyses

### Comparison of compositions with varying time spent between behaviours

**Fig. 1:**
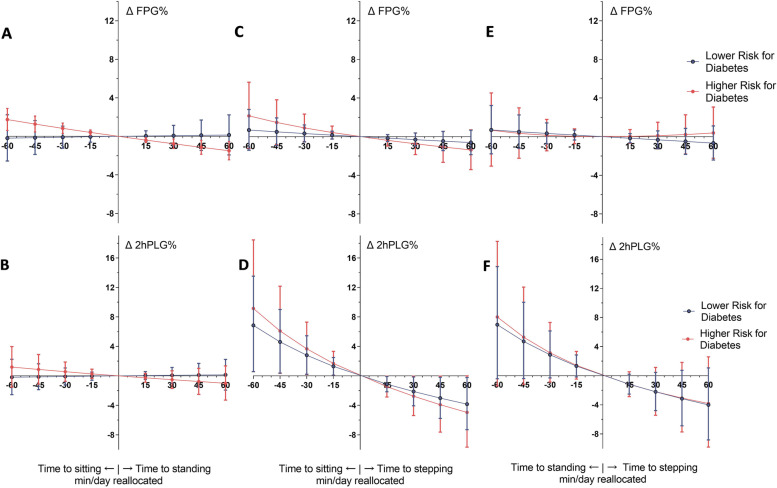
Comparing compositions with varying totals of sitting, standing, and stepping and their associated glucose outcomes in lower and higher risk for diabetes. Graphs **A** and **B** denote compositions with varying time spent sitting (more sitting time and less standing indicated to the left of the x-axis) and standing (more standing time and less sitting indicated to the right of the x-axis) and estimation of fasting, and 2 h plasma glucose dependent on composition. Graphs **C** and **D** denote compositions with varying time spent sitting and stepping. Graphs **E** and **F** denote compositions with varying time spent standing and stepping. The associated glucose outcomes at a given composition are compared to the glucose outcomes at the geometric mean. Difference in glucose between new composition and original are denoted by Δ FPG% and Δ 2hPLG%

New compositions were made by adding and subtracting time from geometric mean values. The new compositions’ estimated glucose values were then compared to original mean values in lower and higher risk. Figure 1 illustrates varying totals of sitting, standing, and stepping, and Table 4 shows 60 min composition variations between two select behaviours.

**Table 4:** Varying composition by 1 h and using linear regression models to estimate glucose

Varying compositions by 60 min^a^	Δ FPG%		Δ 2hPLG%	
	Lower Risk (*n* = 375)	Higher Risk (*n* = 272)	Lower Risk (*n* = 373)	Higher Risk (*n* = 267)
Sit → Sleep	−1.1% (− 2.0, − 0.2)	0.3% (− 1.2, 1.8)	0.9% (− 1.8, 3.6)	1.5% (− 2.1, 5.1)
Sit → Stand	0.0% (−0.8, 0.7)	−1.5% (− 2.4, − 0.5)	0.1% (− 1.9, 2.2)	−1.0% (− 3.3, 1.3)
Sit → Step	−0.6% (− 1.9, 0.7)	−1.4% (− 3.4, 0.7)	−3.8% (− 7.3, − 0.2)	−5.0% (− 9.7, − 0.0)
Stand → Sleep	−1.1% (− 2.2, 0.0)	2.1% (0.3, 3.9)	0.7% (− 2.6, 4.1)	2.7% (− 1.7, 7.3)
Stand → Sit	−0.1% (− 0.9, 0.7)	1.7% (0.6, 2.9)	−0.2% (− 2.6, 2.2)	1.2% (− 1.5, 4.0)
Stand → Step	− 0.7% (− 2.4, 1.1)	0.4% (− 2.2, 3.1)	−4.0% (− 8.8, 1.1)	−3.8% (− 9.8, 2.6)
Step → Sleep	−0.3% (− 2.6, 1.9)	2.5% (− 0.8, 5.9)	7.8% (0.9, 15.1)	10.8% (2.4, 19.9)
Step→ Sit	0.7% (− 1.4, 2.8)	2.1% (− 1.2, 5.6)	6.9% (0.6, 13.5)	9.1% (0.6, 18.5)
Step → Stand	0.7% (− 1.8, 3.2)	0.7% (−3.1, 4.5)	7.0% (−0.4, 15.0)	8.1% (− 1.4, 18.5)
Sleep → Sit	1.1% (0.2, 2.1)	−0.4% (− 1.9, 1.1)	−1.0% (− 3.6, 1.8)	−1.7% (− 5.2, 2.0)
Sleep → Stand	1.1% (0.0, 2.2)	−1.8% (− 3.5, − 0.2)	− 0.8% (− 3.8, 2.3)	−2.6% (− 6.6, 1.4)
Sleep → Step	0.5% (− 1.0, 2.1)	−1.7% (− 3.7, 0.3)	− 4.8% (− 8.8, − 0.6)	−6.5% (− 11.0, − 1.8)

Values expressed as the percentage difference (95% CI) between the new composition’s estimated glucose value and the original geometric mean glucose value (Δ FPG% or Δ 2hPLG%) for each diabetes risk group

^a^Compositions tested varied by 60 min from geometric means, for example, “Sit→ Stand” denotes 60 minutes subtracted from sitting time and added to standing time geometric means

Small differences in estimated fasting glucose outcomes were found when varying the compositions from geometric means. In lower risk, increasing sleeping by 60 min and decreasing sitting by 60 min (Sit→ Sleep) was associated with decreased FPG: –1.1%Δ FPG (95%CI: − 2.0, − 0.2). Lower sitting time, and higher standing time were associated with lower FPG in higher risk only. In higher risk, 60 min more standing, and equivalent less time sitting (Sit→ Stand) was associated with reduced FPG: −1.5%Δ FPG (95%CI: − 2.4, − 0.5). This suggests that standing may be more advantageous for the higher risk group (Fig. 1A). Increased standing time by 60 min, and 60 min less sleeping time (Sleep→ Stand) was associated with greater FPG in the lower risk group (1.1%Δ FPG (95%CI: 0.0, 2.2)) and reduced FPG in the higher risk group (− 1.8%Δ FPG (95%CI: − 3.5, − 0.2)).

The estimated differences in 2hPLG when varying the compositions were greater than in FPG. For example, less stepping, and more sitting time was associated with large differences in 2hPLG from mean values (Fig. 1D) in both risk groups. The greatest estimated glucose differences were observed when increasing sleep by 60 min, and decreasing stepping by 60 min which were associated with similar values in lower risk (7.8%Δ 2hPLG (95%CI: 0.9, 15.1)) and higher risk (10.8%Δ 2hPLG (95%CI: 2.4, 19.9)). While the estimates suggest that the higher risk group had more pronounced differences from the mean, the overlapping confidence intervals equated to no significant difference between risk groups with estimations of Δ 2hPLG.

## Discussion

This is one of few studies to use a postural-based approach to identify sitting, standing, stepping and sleeping time with compositional data analysis methods [28, 29]. We showed that in this sample of middle-aged and older Australian adults, behaviours composing 24 h time-use were associated with biomarkers of glucose control, with some potential differences for those at lower risk and higher risk for diabetes. Compositions that had greater sitting, and lesser equivalent stepping time were most detrimental, however this association did not differ significantly by diabetes risk group. Compositions with greater sitting, and lesser equivalent time standing had small but statistically significant detrimental associations for those with higher risk for diabetes only. These findings may have important practical implications. For example, a person at higher risk of diabetes may improve glycaemic control not only with physical activity, but with greater levels of standing time (albeit more modestly so), which can be achieved over the course of the 24 h day.

Interestingly, compositional modeling showed the increase to 2hPLG levels with 60 min more sitting (lower risk: + 6.9% 95CI%: 0.6, 13.5; higher risk: + 9.1% 95CI%: 0.6, 18.5) outweighed the attenuation of 2hPLG with 60 min more stepping (lower risk: −3.8% 95CI%: − 7.3, − 0.2; higher risk: −5.0% 95CI%: − 9.7, − 0.0), indicating a potential ceiling effect of benefit from daily stepping. Similar asymmetric relationships have been observed in compositional analyses before [9, 10, 29, 30], and previously hypothesised to be either relevant to the outcomes and behaviours observed, or to be inherent to the compositional data. For the present findings, it may suggest that higher daily sitting deconditions glycaemic control at greater levels than what is achieved by stepping to improve it. The relationship of time-use and glycaemic control should be investigated further to confirm these findings.

Standing was inversely associated with FPG in the higher risk group only. Whilst there is evidence of standing being beneficially associated with mortality [31–33], many investigations purport that most, if not all glucose change is induced by reallocating time to movement [6, 29, 34], potentially as standing produces only marginal increments in muscle activity and energy expenditure [28]. The reason why the findings are exclusive to the higher risk group is unclear. However, previously standing has been demonstrated to reduce glucose levels in an overweight and obese cohort [12], and chronic muscular inactivity is more likely to induce hepatic insulin resistance in those with familial predisposition for type 2 diabetes [35], indicating that the finding may be inherent to the group studied. Future studies should investigate the extent to which standing, without ambulation is associated with beneficial metabolic outcomes, and clarify whether there is greater benefit to those more vulnerable to chronic diseases such as people with metabolic impairment or at higher risk of type 2 diabetes.

Recent experimental findings have reported that the most exaggerated postprandial glucose responses to prolonged sitting are evident in those with poorer underlying glycaemia, and insulin resistance [16]. When skeletal muscles are inactive, GLUT4 expression is down-regulated [36]; this leads to less contractile-mediated glucose uptake and more circulating glucose in the bloodstream. Being at higher risk for diabetes may increase susceptibility to insulin resistance, thus impairing the action of insulin mediated glucose disposal [37]. Therefore, those at higher risk for diabetes may have diminished capacity for homeostatic control of glucose when exposed to high volumes of sitting during the day. Considering mean estimates only, those with higher risk did exhibit a propensity for greater levels of 2hPLG with compositions higher in sitting and lower in stepping, and greater relative attenuation of 2hPLG levels with compositions higher in stepping and lower in sitting when compared to the lower risk (Fig. 1D). However, the overlapping confidence intervals suggest no significant difference by risk group for these relationships. Interestingly, compositions with higher stepping, and less sleeping were associated with reduced 2hPLG too. Evidence outlining favorable sleep duration supports 7 – 8 h per day, and sleep time that extends beyond this duration is associated with less beneficial health outcomes [11]. Given the mean sleeping period for both risk groups was > 8.4 h per day, it is not unfeasible that excessive sleep durations, especially in place of daily physical activity, may be less favourable for glycaemic control. These findings however should be interpreted with caution given the cross-sectional study design, where reverse causation (i.e. worsened metabolism provokes longer sleep durations) cannot be ruled out. Future studies incorporating larger sample sizes, and a longitudinal study design should investigate these findings further.

Compositions that had 60 min more stepping time and equivalent reduction in sitting time had comparable associations with glucose outcomes to those that have previously been reported in a physical activity intervention. Gong et al. [38] determined that physical interventions achieved significant 2hPLG reductions when comparing the standardised mean differences to controls (SMD: −0.42; 95% CI: − 0.63, − 0.20). The results of the present study are comparable, albeit of lower magnitude, for both lower (SMD: −0.16 95%CI: −0.19, − 0.13) and higher risk (SMD: −0.13 95%CI: − 0.15, − 0.10) when comparing more physically active compositions to the original geometric mean composition. Difference in magnitudes may be explained by physical activity interventions specifically involving participants in more intensive, and controlled experimental approaches as opposed to the free-living context of the present study.

Recent public health and clinical practice guidelines now emphasise the importance of not only reducing total sedentary time, but also increasing total physical activity through moving more throughout the day – ‘sit less and move more’ [3]. Our findings suggest that for glucose outcomes, this approach may be of greater importance in those at higher risk for development of diabetes, which is aligned with landmark diabetes prevention trials primarily targeting those at elevated risk [39]. A recent systematic review by Hadgraft et al. [40] evaluated a large sample of sedentary behaviour change interventions and determined only small benefit of reducing sedentary time on FPG. Given the findings of the present study, the small benefit to glucose levels may be explained by the majority of included studies recruiting the general population (therefore not necessarily at high risk of chronic disease), as well as having a degree of heterogeneity between intervention components and intervention messaging. Notably, most of the intervention trials in the systematic review resulted in increased standing behaviours only, as opposed to changes to stepping levels. Future interventions should target the replacement of sedentary behaviour with both standing and stepping behaviours, and target those at elevated risk of diabetes.

The main strengths of this study were the measurement of 24 h posture time-use with device-based measures in the free-living context, as well the interpretation of this with a CoDA technique. The participants were recruited from the general Australian population, indicating that these findings may apply to a broader population, however it should be noted that this study’s subsample of participants were on average healthier than the main sample [7]. By using the activPAL device it was possible to accurately interpret postures such sitting, standing, and stepping, which has rarely been applied to a cohort at risk of diabetes. However, findings do need to be considered in the context of the limitations. Notably, cross-sectional analyses preclude causal inference. Another limitation is that selection bias may be present as stratification by diabetes risk group at 39 mmol/mol HbA1c may lead to reduced glucose outcome variability (and a greater chance of type 2 error); however there were similar estimates with 2hPLG between groups for sitting and stepping. The CoDA modeling did not account for patterns of time (e.g. short or long periods of sitting /or stepping), intensity (light, moderate, or vigorous intensity), nor sleep quality, which are known to have independent associations with cardiometabolic biomarkers [8, 41, 42]. There may have been some errors in the sleep estimate as sleep onset was self-reported or automatically estimated rather than objectively measured.

## Conclusion

In conclusion, these findings from an examination of 24 h time-use (composed of sitting, standing, stepping and sleeping) in participants going about their normal daily lives, suggest that in middle-aged and older adults, glycaemic control could be improved by reducing daily sitting time and supplementing it with standing or stepping, especially so in those at higher risk of developing type 2 diabetes.

### Tables

See Tables 1, 2 and 3.

## Data Availability

Datasets are available on request to corresponding author.

## Abbreviations

2hPLG: 2 h post-load glucose
AusDiab: The Australian Diabetes, Obesity and Lifestyle Study
CoDA: Compositional data analysis
FPG: Fasting plasma glucose
HbA1c: Glycated Haemoglobin
ilr: Isometric log ratio
OGTT: Oral glucose tolerance test
VIF: Variance inflation factor
